# State of the Art on Green Route Synthesis of Gold/Silver Bimetallic Nanoparticles

**DOI:** 10.3390/molecules27031134

**Published:** 2022-02-08

**Authors:** Angela Scala, Giulia Neri, Nicola Micale, Massimiliano Cordaro, Anna Piperno

**Affiliations:** 1 Department of Chemical, Biological, Pharmaceutical and Environmental Sciences, University of Messina, V.le F. Stagno d’Alcontres 31, 98166 Messina, Italy; ascala@unime.it (A.S.); giulia.neri@unime.it (G.N.); nmicale@unime.it (N.M.); mcordaro@unime.it (M.C.); 2 CNR-ITAE, Via S. Lucia sopra Contesse, 5, 98126 Messina, Italy

**Keywords:** bimetallic nanoparticles, noble core-shell nanoparticles, plasmonic materials, green nanotechnology, noble metal alloy nanoparticles

## Abstract

Recently, bimetallic nanoparticles (BMNPs) blending the properties of two metals in one nanostructured system have generated enormous interest due to their potential applications in various fields including biosensing, imaging, nanomedicine, and catalysis. BMNPs have been developed later with respect to the monometallic nanoparticles (MNPs) and their physicochemical and biological properties have not yet been comprehensively explored. The manuscript aims at collecting the main design criteria used to synthetize BMNPs focusing on green route synthesis. The influence of experimental parameters such as temperature, time, reagent concentrations, capping agents on the particle growth and colloidal stability are examined. Finally, an overview of their nanotechnological applications and biological profile are presented.

## 1. Introduction

In recent years, the design and synthesis of noble metal nanostructures have been gaining tremendous interest owing to their unique size- and shape-dependent properties. They have been proposed for applications ranging from electronic and biomedical devices to environment and energy technologies [1,2,3,4,5]. Moreover, the intrinsic biological properties of silver and gold nanoparticles (Ag NPs and Au NPs) as antimicrobial, anticancer, and anti-leishmanial agents have been investigated [6,7,8,9]. As compared to the monometallic nanoparticles (MNPs), the bimetallic nanoparticles (BMNPs) constitute a new class of nanostructures that generally displays superior technological relevance. The properties and the applicability of the BMNPs not only depend on their size and shape but also on their metal composition and fine structure (i.e., alloyed structure, core-shell, hetero-structured). 

The modern scientific evaluation of MNPs and BMNPs originated from the pioneering research of Michael Faraday that explored the preparation of colloidal Au solutions by reducing Au salts with phosphorus in water [10], and the fundamental studies by Enüstün and Turkevich [11] that reported the preparation of spherical Au NPs and Ag NPs with tunable sizes by reduction of Au or Ag salts using citrate in aqueous solutions. The early investigations on BMNPs were pioneered by Morriss and Collins, which synthesized Au@Ag BMNPs in core-shell architecture in the 60s [12]. 

To obtain Au/Ag BMNPs in high yields, good uniformity, well-defined geometries, the control over their growth during the synthesis is highly requested. The main synthetic strategies include: (i) continuous growth; (ii) seed-mediated growth; (iii) galvanic replacement reaction (GRR). These chemical procedures can be combined with each other for the preparation of more complex metal nanostructures. The continuous growth strategy entails the co-reduction of chloroauric acid (HAuCl_4_) and silver nitrate (AgNO_3_) in the presence of a reducing agent. These co-reduction of metal ions in the presence of a stabilizing agent (e.g., sodium citrate) in boiling water, (known as Turkevich method) [11] llows the formation of alloy BMNPs, although some literature data also indicated the possible formation of core-shell nanostructures [13]. The presence of a single LSPR (Localized Surface Plasmon Resonance) band in the optical absorption spectra indicated the co-reduction of both metal ions with the formation of homogeneous Au-Ag alloy. Furthermore, the increase of Ag content in the BMNPs resulted in a linear blue-shift of LSPR band [14]. Two separate plasmonic bands are usually expected for core-shell nanostructures, although a morphology with a sufficiently thick shell may also lead to a single band. Composition and size controllability of alloy BMNPs are challenging due to the propensity of silver ions to induce precipitation with chloride ions from HAuCl_4_. Therefore, to avoid AgCl precipitation, suitable dilution conditions should be adopted [14].

The seed-mediated growth protocols require the preparation of the first metal component of the binary nanosystem and the subsequent nucleation and growth of the second component on the first metal. According to this strategy, core-shell or hetero-structured BMNPs can be obtained. The growth of the second metal component strongly depends on the preformed seeds and proper reaction conditions (i.e., capping agent, temperature, and pH). Due to straightforward reduction of gold ions into their low potential form, different types of Au NPs have been investigated as seeds for the Ag deposition. The morphology/shape of the Au/Ag BMNPs is affected by the reaction parameters (i.e., temperature, pH, time, reagent concentrations, ligands) regardless of whether spherical Au NPs are used as seeds. The presence of ligands plays a crucial role in the seed-mediated growth strategy since during the deposition of the second metal component on the first, a dynamic adsorption/desorption of the ligands, can occur on the seed surface, affecting the growth of underlying facets. Facets with preferred ligand binding are less exposed and therefore grow slower [15]. Feng et al. reported that the morphology of Au/Ag BMNPs could be tuned from concentric core-shell, eccentric core-shell, and acorn, to dimer structures by simply varying ligand conditions during the growth of Ag on Au seeds [16].

The electrochemical process that involves the oxidation of a sacrificial metal template by ions derived from another metal owning a higher reduction potential is known as galvanic replacement reaction (GRR). If Ag NPs are used as seeds for Au overgrowth, BMNPs with an Ag@Au core-shell structure cannot be fully obtained and will be replaced by hollow and porous Au/Ag alloy BMNPs. The first example of this pioneering strategy was described by Xian et al. for the preparation of hollow Au/Ag BMNPs from Ag NPs and has been successfully extended to many other metals [17,18].

**Figure 1:**
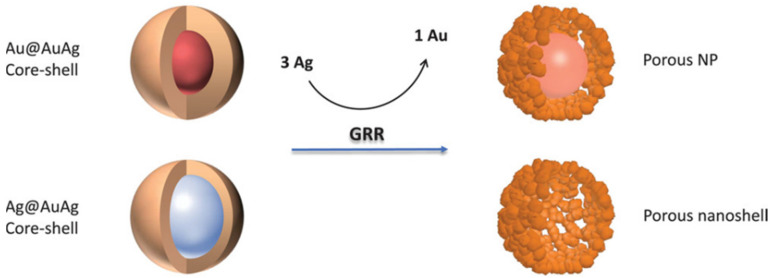
GRR on Au@Au/Ag and Ag@Au/Ag, resulting in porous shell with solid core (porous NP) or hollow interior (porous nanoshell), respectively. Reprinted from Reference [19].

The GRR of Ag NPs in the presence of HAuCl_4_ is depicted in Figure 1 [19]. The driving force of this phenomenon is the favorable difference in electrochemical potential between Ag^+^/Ag (0.80 V) and AuCl_4_^−^/Au (1.00 V) [20]. 

The regular and ordered distribution of metal atoms in BMNPs is fundamental for the optimization of their physicochemical properties. Therefore, each preparation procedure must be aimed at the controlled and reproducible synthesis of the target BMNPs. It is well known that the synergy between different metal atoms is an indispensable factor for improving the stability and applications of the resulting BMNPs [18]. Taking into consideration all these requisites, the research in this field has turned toward wet-chemistry based preparation techniques that make use of non-toxic and low-cost chemical reagents. The synthetic procedures were optimized in order to modulate the features of BMNPs according to the desired applications. Following this goal, the use of environmentally friendly protocols such as the use of plant extracts, biomolecules, and biopolymers has gained increasing interest. They can be considered ideal reagents as they meet all the required conditions of biocompatibility and accessibility, but they also have multi-purpose action as reducing, capping and shape-directing agents.

The review deals with the recent advancements of green synthesis procedures for the preparation of Au/Ag BMNPs. We will discuss, using literature explicative examples, the main features of these BMNPs together with their main nanotechnological applications and their biological profile. Until now, a clear nomenclature to define the different fine structures of the BMNPs according to their metal composition (i.e., alloyed structure, core-shell, hetero-structured, etc.) has not yet been adopted. In this review, then, the terms Me@Me, Me-Me and Me/Me indicate a core-shell architecture, alloyed structure and not defined composition, respectively. Moreover, in the discussion of the protein-based green synthesis procedures, a brief paragraph is devoted to nanoclusters (NCs), molecular assemblies that consist of a few to a hundred atoms [21].

## 2. Green Synthesis Procedures for Noble BMNPs Preparation 

Synthetic methods for the preparation of BMNPs can be classified in two main strategies depending on the approaches followed to produce the nanostructures; namely, the bottom-up method and the top-down method. The first method (or constructive process) uses atoms or molecules as precursors, which react to form the BMNPs. The most used techniques include the use of sol-gel, chemical vapor deposition, molecular self-assembly, atomic layer deposition, nanocluster sources, and wet-chemical reduction. The second procedure (or destructive method) entails the employment of bulk materials that are finely pulverized into nanometer-sized particles or modeled into nanostructures. 

Recently, the main principles of green synthesis have been introduced into the methods of generation of BMNPs. Specifically, the biocompatibility of the substances used to produce the nanomaterials has been considered and thoroughly reviewed [22,23]. A series of the most representative examples of preparations of BMNPs, including biogenetic synthesis and the protocols based on the use of macrocycles, carbohydrate polymers, proteins, and so forth, are summarized in Table 1 and herein discussed.

Biogenic syntheses (photosynthesis) using plant extracts, are in continuous development. However, the protocols of these synthetic procedures should be reviewed and classified to provide the optimization criteria necessary for the design of BMNPs with reproducible features. The phytosynthesis of BMNPs first involves the preparation of aqueous extracts of plants, such as leaves fruits, flowers, seeds, and roots. The aqueous extracts are then mixed with the metal reagents and the reaction generally proceeds at mild temperature, i.e., 70–80 °C. The reduction of the metal ions and the stabilization of the metal nanostructures are favored by the synergistic action of various phytochemicals (e.g., phenolic acids, flavonoids, terpenoids, alkaloids, and carbohydrates) containing functional groups such as carboxylic acid, carbonyl, hydroxy, and amino groups.

Alti et al. reported the green synthesis of alloy Au-Ag BMNPs by a single-step reduction process using fenugreek, coriander, and soybean leaf extracts [24]. The synthetic procedure was performed by boiling the mixture of leaf extracts with HAuCl_4_ and AgNO_3_ solutions at 80 °C for 30 min. The transformation of the colorless solution into a red color solution indicated formation of the alloy Au-Ag BMNPs. 

K. Muthu et al. recently published a standard procedure of ultrasound-assisted green synthesis of alloy Au-Ag BMNPs from *Lawsonia inermis* seed extract aqueous fraction. In a typical procedure the Au-Ag BMNPs synthesis was carried out by mixing the metal precursors solution (HAuCl_4_ 1 mM and AgNO_3_ 1 mM, 1:1 ratio) to *Lawsonia inermis* seed extract aqueous solution under sonication at room temperature for 10 min. The color of the reaction mixture changed from pale yellow to dark purple [25]. 

Lomelí-Marroquín et al. described the synthesis of colloidal starch-stabilized Ag-Au alloy BMNPs. The reaction was carried out by adding the Au/Ag metal precursor solution to starch solution at 70 °C and pH = 11 [26]. Glucose units, released during the prolonged heating of starch at high temperatures (~95 °C) and basic conditions, were oxidized to gluconic acid (standard reduction potential [E°] at pH ≈ 10 of −0.60 V) and the precursor ions Ag^+^ (Ag^+^/Ag°, E° = +0.80 V) and AuCl_4_^−^ (AuCl_4_^−^/Au°, E° = +1.00 V) were reduced to their corresponding elemental forms [26]. The BMNPs were stabilized by formation of inter- and intramolecular hydrogen bonding with hydroxyl groups of starch. The expected co-reduction of Ag^+^ and AuCl_4_^−^ ions by glucose molecules with the resulting formation of AgCl as a by-product due to the galvanic replacement reaction between Ag(s) and AuCl_4_^−^ was not observed [35]. 

Ag-Au alloy BMNPs coated with starch were employed as seeds for the preparation of anisotropic Au-based nanostructures (Au-ANs) using H_2_O_2_ as green reducing agent. Hydrogen peroxide may be considered a “green” reagent because water and oxygen are the only by-products [27]. The growth of Au-ANs occurred under kinetic conditions by setting a relatively low temperature (10–15 °C) and a neutral pH (∼7) to tune the reactivity of H_2_O_2_ and to control the reduction rate of Au^3+^ to Au^0^. Morphological analyses revealed the coalescence of metal NPs generating a collection of dimers and trimers; UV-vis analyses revealed two distinguishable signals related to the transversal and the LSPR modes. The transversal LSPR band, with local λ_max_ at 522 nm, is associated with the shorter axis of the anisotropic nanostructure, whereas the longitudinal LSPR band, with λ_max_ around 702 nm, is attributed to the coupling along the interparticle axis. 

**Figure 2:**
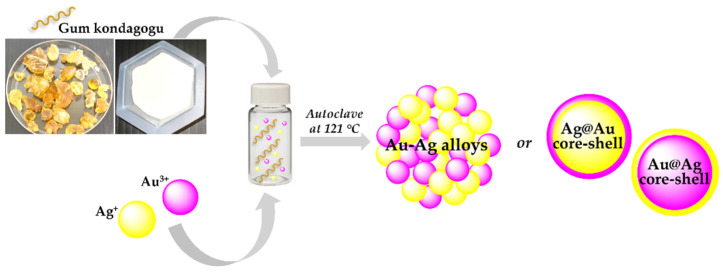
Schematic representation of bimetallic nanoparticles synthesis, using green approach and the formation of possible nanostructure.

Ag/Au, Ag/Pd and Au/Pd BMNPs were prepared using the natural kondagogu rubber (GK) biopolymer as a reducing and capping agent [28]. In the adopted procedure, the colloidal GK solution (1.0%), containing the metal ions (1 mM) for each metal (1:1 molar ratios), was autoclaved (121 °C; 15 psi) for 15 min using a laboratory-grade autoclave. TEM and UV-vis analysis indicated the formation of monodispersed spherical BMNPs due to the reduction of metal atoms by electron transfers between the ionic metal and hydroxyl or aldehyde groups in GK. UV spectra suggested the formation of different nanostructured architectures (Figure 2) according to the metal precursors and the adopted experimental conditions. The sequential reduction of silver, gold, and palladium atoms produce heterogeneous bimetallic alloys (Au@Ag, Ag@Pd, and Au@Pd), and simultaneously, the unreacted second different metal present in the aqueous reaction system may react to form alloy or core-shell nanomaterials at higher temperatures. 

The catalytic activity of BMNPs was evaluated in the reduction reaction of 4-nitrophenol (4-NP) to 4-aminophenol by NaBH_4_. The performed studies showed that the catalytic efficiencies of three bimetallic nanocomposites followed this order Ag-Pd > Ag-Au > Au-Pd. 

In a recent paper, Y. Shkryl et al. described a rapid approach for synthesis of Ag/Au BMNPs using the callus extract of *Lithospermum erythrorhizon* (BK-39) able to function as reducing and capping agent, because it is abundant in polysaccharides and polyphenols. The preparation procedure involved mixing the AgNO_3_ and HAuCl_4_ solutions with the extract of BK-39 and continuous irradiation of light and subsequent separation of the BMNPs by centrifugation [29]. Different Ag:Au ratios (i.e., 4:1, 1:1, and 1:4) as function of reaction time were investigated. It was reported that the rate increase of the Au NPs LSPR band (λ_max_ = 545 nm) was much higher than that of the Ag NPs LSPR band (λ_max_ = 440 nm). Consequently, the Ag NP absorbance peak was not visible even in the case of Ag/Au (4:1). The obtained UV-Vis spectra, displaying mainly one well-distinguishable LSPR peak positioned at the same wavelength as the Au NPs, suggested a core-shell structure with gold on the shell.

**Figure 3:**
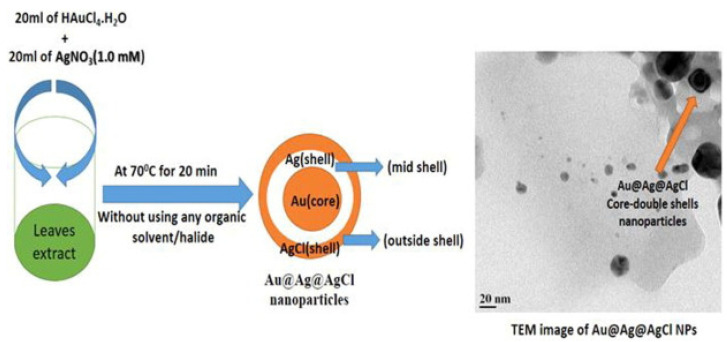
Schematic representation for the formation of Au@Ag@AgCl core/double shell NPs. Reprinted from Reference [30].

The formation of the core-double shells Au@Ag@AgCl BMNPs employing medicinal leaves extract was reported [30]. The triple layer BMNPs were prepared by heating the mixture of HAuCl_4_/AgNO_3_ (4:1 and 1:4 M ratios) and 10% *Momordica charantia* leaves extract (Figure 3) at 70 °C for 20 min. TEM image displayed an inhomogeneous sample where some Au@Ag@AgCl BMNPs can be appreciated (see detail in Figure 3). 

**Figure 4:**
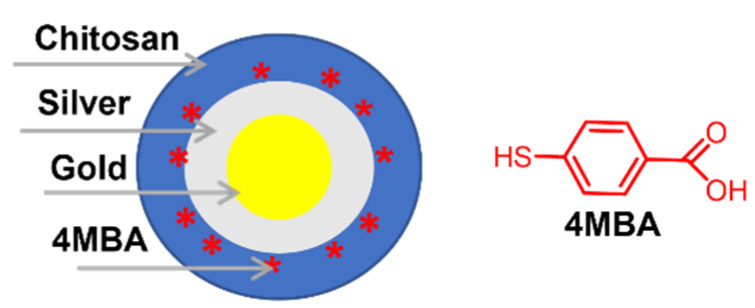
Schematic representation of Au@Ag BMNPs coated with chitosan and labelled with 4MBA.

Chitosan-coated Au NPs were used as core materials to fabricate reproducible, stable, and biocompatible Au@Ag core-shell BMNPs enveloped in a chitosan layer. The synthetic protocol reported by Hada et al. [31] includes, in the first step, the preparation of chitosan-coated Au NPs as a seed material, and, in the second step, the growth of an Ag shell by three subsequent additions of silver ions. The treatment of Au@Ag BMNPs at room temperature with 4-mercaptobenzoic acid (4MBA) solution produced the surface enhanced Raman scattering (SERS) labelled nanoTag (Figure 4) that was investigated for cell imaging under scanning confocal Raman microscopy. 

**Figure 5:**
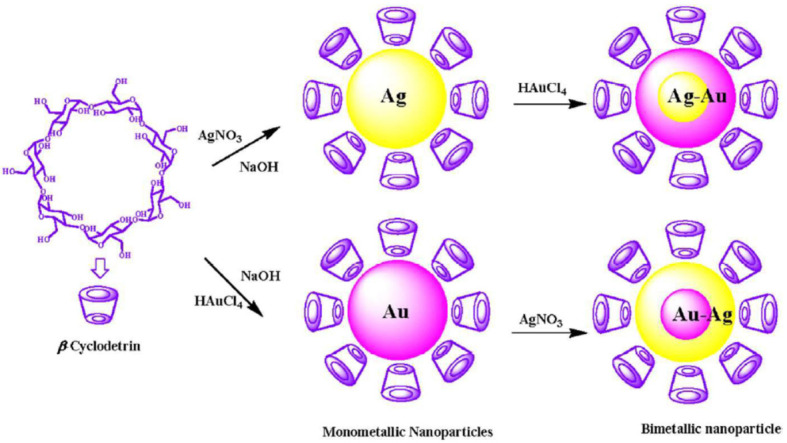
Schematic representation of MNPs and BMNPs using CDs. Reprinted from reference [32].

Different cyclodextrin (CDs) were investigated as both reducing and stabilizing agents for the green preparation of Au@Ag BMNPs and of inverted core-shell Ag@Au BMNPs (Figure 5). The synthetic procedures were carried out in aqueous mildly alkaline medium, and the reaction rate depended on concentration of metal precursor, CD concentration, and temperature. The authors reported that the reaction started immediately after mixing the metal salt solution with alkaline CD solution. Within 20 minutes, the colorless solution of the silver salt converted first into a yellow color, then into a violet-pink color. These two colors indicate the characteristic surface plasmon absorption of the silver and gold NPs, respectively. The authors reported that, at these experimental conditions, silver and gold ions are reduced by the hydroxyl groups of CDs since each β-CD molecule has seven primary hydroxyl groups and 14 secondary hydroxyl groups. The atomic percentage of silver is less than gold in Ag@Au core-shell NPs and the atomic percentage of gold is more that silver in Au@Ag core-shell NPs even if the same amount of metal ions was used for both the reaction steps.

Fierascu et al. reported the phyto-mediated synthesis of Au@Ag nano-architectures by co-reduction of silver and gold ions using the ethanolic extract of *M. officinalis* [33]. Biomolecules of extract acted as both reducing and stabilizing agents with the (poly)phenolic component most likely involved in the metal reduction processes. The authors suggested the formation of a core-shell architecture with a coating of Ag around the Au NPs on the bases of silver and gold plasmon bands detected in UV-vis spectra. 

**Figure 6:**
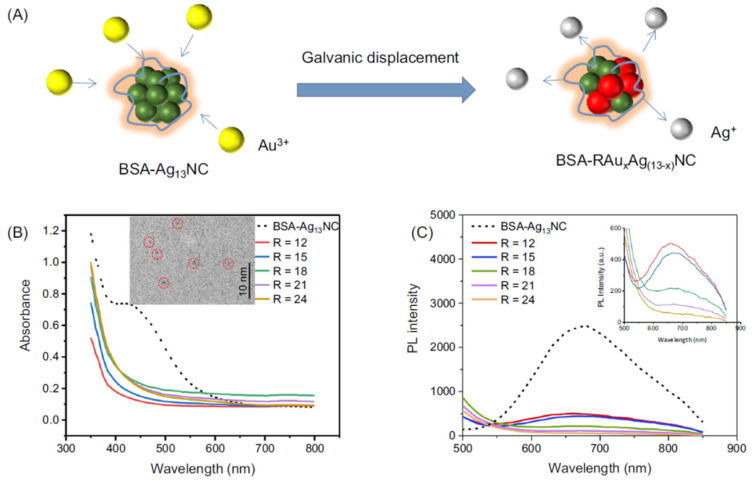
(**A**) Schematic illustration of the BSA-RAuxAg(13 − x)NCs formed by GRR. (**B**) UV-vis and (**C**) photoemission spectra (λex = 480 nm) of BSA-protected Ag13NC (dashed lines) and BSA-RAuxAg(13 − x)NCs (solid lines) where R indicates the molar ratio of Au ions to AgNC. (Inset of B) TEM image of BSA-24AuxAg(13 − x)NCs. The red circles highlight the individual alloy nanoclusters. (Inset of C) Enlarged photoemission spectra of BSA-RAuxAg(13 − x)NCs for R = 12, 15,18, 21 and 24 respectively. Reprinted from reference [34].

Proteins are considered excellent biotemplates for the synthesis of metal nanomaterials due to their unique chemical structures that provide multifunctional active binding-sites to mediate the nucleation and the direct synthesis of a large range of inorganic nanostructures, including Ag/Au nanoclusters (NCs) and Ag/Au BMNPs [36]. NCs are molecular assemblies that consist of a few to a hundred atoms. The sizes (about 2 nm) are comparable to the Fermi wavelength of electrons, which endows them with an important role-the missing link between single metal atoms and plasmonic metal NPs [21]. Bovine serum albumin (BSA) was widely exploited as protein scaffold for NCs synthesis. It was reported that BSA can produce highly stable Au NCs under mild conditions and without the adding of external reducing agents. BSA Tyr residues can act as electron donors to reduce gold ions, while Cys residue stabilize Au NCs by the formation of Au−S chemical bonds. Yua et al. synthetized ultrasmall Au/Ag alloy nanoclusters (AuAgNCs) by reaction of different amount of gold atoms into the BSA-protected Ag13NC (13 Ag atoms/cluster) via a thermodynamically favorable galvanic displacement reaction at room temperature (Table 1, Figure 6). 

## 3. Structural Features of BMNPs

The full characterization of BMNPs, which is a crucial step towards understanding their formation and a prerequisite for any applications, comprises not only the determination of the classical parameters that are required to characterize MNPs (i.e., size, size distribution, shape, crystallographic nature, surface functionalization, and charge), but also further information concerning the overall elemental composition, the ratio of the two metals and the internal distribution and homogeneity of the elements in individual NPs [37]. Ideally, these parameters should be determined by “non-invasive” time-resolved techniques, directly applicable in the reaction medium.

The typical method is the UV-Vis-IR absorbance spectroscopy. However, useful information can be also provided by the small-angle X-ray scattering (SAXS). 

Transmission electron microscopy (TEM) and inductively coupled plasma mass spectrometry (ICP-MS), requiring extracting the particles from their environment, are generally used to validate the information [38]. Among these four complementary methods, SAXS yields the most information about the NPs, such as the size, polydispersity, shape and number density. Actually, scattering methods only provide indirect information, whereas imaging techniques remain essential for identifying the particle shapes; therefore, TEM analysis should always be used as a complement. Furthermore, the ICP-MS is a very robust technique yielding the elemental concentration in solution and the Ag/Au ratio within the nanoparticles, without providing information regarding size, shape and polydispersity of the objects [38]. 

Depending on the elemental composition, BMNPs have peculiar optical properties that are different from both the bulk metals and the MNPs [39]. Specifically, LSPR depends on surface electrons that are excited to give a characteristic absorption, leading to colored nanoparticle dispersions. The wavelength and intensity of the LSPR band and the color of the solution depend on particle size, shape, environment, number density, and nanostructures arrangement. As an example, dispersions of Ag NPs with a diameter of about 10–20 nm have a yellowish-brown color, whereas the same-sized Au NPs dispersions typically have a red color. Dispersions of larger-sized Au NPs, instead, are blue [39]. The LSPR of BMNPs can give a clue about the internal distribution of the elements. In alloyed NPs, the maximum of the LSPR absorption band shifts linearly with the composition, whereas in core-shell NPs, the LSPR absorption is observed only for the outermost metal together with a typical dark-violet brownish color [37]. 

J. Wilcoxon investigated how the type of nanostructures (core-shell vs. alloy configurations) influences their absorbance features and optical responses, pointing out a very weak dependence of plasmon energy and damping in nanoalloys compared to core-shell structures of equal size, even when they have the same Au and Ag contents [40]. 

Actually, the optical properties of core-shell BMNPs and the profile of the resonance bands of a gold nanoparticle in contact with a silver metal depend on the composition and distribution of each metal and also by the thickness of shell. Gold nanosphere band is generally localized toward ≈516 nm; this band is observed at ≈400 nm for silver nanosphere, whereas a shift to ≈437 nm in the synthesis of BMNPs indicates a lower percentage of gold outside compared to silver, attributable to an Au-core/Ag-shell nanostructure [41]. 

Plasmonic nanostructures hold great potential in enhancing fluorescence emission. In this regard, core-shell BMNPs can be properly functionalized with fluorophore labels at a precise distance from the NP surface to obtain fluorescence enhancement and to avoid metallic quenching. It is well known that the presence of metals near fluorescent dyes improves their photophysical properties, increasing fluorescence signals due to near-field photophysical interactions. This phenomenon is called metal-enhanced fluorescence (MEF). Recently, multiple research groups have shown interest in the development of plasmonic BMNPs designed to exploit the advantages of MEF over commonly used fluorescent molecules and used as contrast agent for multiplexed cell-imaging application [42]. 

## 4. Nanotechnological Applications of BMNPs

The current interest in Au/Ag BMNPs is growing in catalytic, electrochemical, and sensing fields due to the improved optical, thermal, and electrical properties of these dual nanosystems as compared to the monometallic counterparts. Control of the size, shape, chemical composition, and structure is crucial for these applications to get better performances [43,44]. Good catalytic activity is ascribed both to a greater surface area and an alignment of the work function or electronic levels of the two metals, which enhances the electronic charge shift [45,46]. 

The catalytic activity of Au/Ag BMNPs prepared via seed colloidal technique were evaluated by using 4-nitrophenol (4-NP) reduction as a model reaction, in presence of NaBH_4_ [47]. Au/Ag BMNPs catalyzed the reduction process by facilitating the electron transfer step from the donor BH_4_^−^ to the 4-NP acceptor. The authors described a faster reaction rate in the presence of BMNPs compared to Au NPs, probably due to the synergic effect between the two metal elements which leads to an enhancement of the electron density on the bimetallic surface. Moreover, an increase of the surface area with small sizes also improved their catalytic performance. Comparable results were reported by Dsouza and co-workers which described a 97% conversion of nitro to amino aromatic compound in 8 min by using Au/Ag BMNPs [48]. Ag@Au BMNPs prepared by a green procedure employing a polysaccharide extracted by mushroom *Ramarai botrytis* were also shown to possess high catalytic ability and to achieve a complete reduction of 4-NP to 4-aminophenol [49]. 

Tremel et al. reported an accurate study on the preparation of segregated or alloyed Au, Ag and Au@Ag NPs using plant extracts of *Pulicaria undulata* as a reducing and stabilizing agent. This study underlined a high reduction efficiency and the dependence of the plant extract concentration, both on the rate of formation and on the morphology and composition of the resulting BMNPs. Well-defined BMNPs were obtained at high concentrations of plant extract and were used as catalysts in the reduction reaction of 4-NP with NaBH_4_. The efficiency of the nanocatalysts was correlated to the well-tuned morphology, as a larger active surface area of the BMNPs improves the interaction capacity of the active site [50].

The catalytic activity in the oxidation process of lactose (LA) to lactobionic acid (LB) in aqueous solution of Au/Ag BMNPs supported on Al_2_O_3_ was investigated [51]. The nanostructure and catalytic activity of the bimetallic Ag/Au/Al_2_O_3_ strongly depends on the Au/(Au + Ag) ratio. For an atomic ratio of 0.5 or lower, core-shell BMNPs were obtained with the external Ag layer that completely covered the Au core. These BMNPs showed extremely week catalytic activity. The BMNPs with a ratio higher than 0.5 showed a crystalline structure with Au-Ag content homogeneously distributed and enriched with Ag on the surface. The best catalytic activity was observed with an atomic Ag/Au surface ratio close to 1. It is likely that a cooperative effect between silver and gold atoms occurred: Ag chemisorbed and activated O_2_ molecules, while Au interacted with the carbonyl group of LA and the closeness of the metallic sites improved the LA/O_2_ interactions in the oxidation process. This cooperative effect is not allowed in the BMNPs with a core-shell structure. 

Another reason for the success of Au/Ag BMNPs concerns their excellent optical properties. The optical characteristics of the core-shell BMNPs, such as LSPR peaks at λ_max_ and intensity of absorption spectra, are crucial in designing multifunctional nanoparticles for wide-ranging applications [52]. George et al. prepared an optical and electrochemical sensor to detect Mn(II) ions and Ciprofloxacin (CIP). β-CDs were used as capping and reducing agent to synthetize Au/Ag BMNPs by microwave treatment in water medium. β-CDs promoted the interactions between BMNPs and Mn(II) ions or CIP, which resulted in a change of the color solution and in surface plasmon resonance in optical sensing measurements. To test the performance as electrochemical sensor, the Au electrode was modified with Au/Ag BMNPs by drop casting method and cyclic voltametric studies were carried out. The added Mn(II) ions bound the BMNPs of the Au electrode and the complexation induced an enhancement of the redox current on the electrode surface, which resulted in an increase of the redox peak together with a negative shift in the reduction peak current. The electrochemical detection of CP was possible thanks to its affinity towards β-CD cavity and the good electrical conductivity of the Au/Ag BMNPs. The increase of the oxidation peak current after CIP addition is due to an electrochemical oxidation of CIP pointing out an irreversible electrode process. Tests performed in the presence of other competing metal ions indicated a high detection sensitivity of this sensor. In particular, a detection limit of 8.42 and 18.40 nM for Mn(II) and of 10.26 and 7.24 nM for CIP were obtained by optical and electrochemical approaches, respectively. 

An electrochemical sensor based on screen printed carbon electrode modified with Au/Ag oxides BMNPs for the direct detection of Cr(III) ions was reported by Zhao et al. [53]. At the same time, Cr(IV) ions were revealed by an Au/Ag BMNP based sensor. The two systems showed a linear range of 0.05–5 and of 0.05–1 ppm and a detection limit of 0.1 ppb for Cr(VI) and Cr(III), respectively. In both cases, the intensity signal generated by BMNP electrode was twice as high compared to Au or Au oxides-based electrode, whereas no oxidation peaks were detected for Ag or Ag oxides-based electrode, proving an absence of Ag role in the electrode reaction. The two sensors were able to detect chromium species also in the real wastewater and the authors speculated about their combination in a dual-channel electrochemical device to reach new *in-situ* chromium detection tool. 

Core-shell Au@Ag BMNPs showed significant advantages in comparison to both Au and Ag NPs as Raman active substrates. Specifically, most of the LSPR bands of BMNPs are in the visible region, clearly indicating that these nanostructures are good candidates for SERS application [54]. Interestingly, Au-Ag BMNPs combine the good properties of both metals, as Ag is more effective than Au in terms of plasmonic enhancement, whereas Au provides more chemical stability [54]. The high stability and possibility to tune the size and composition, together with the excellent optical properties and signal enhancement, make them promising SERS detection devices. 

Hussain et al. developed core-shell Au@Ag BMNPs as Raman active substrate to simultaneously detect two hazardous chemicals contaminants—dicyandiamide and thiram—in milk samples [55]. A detection limit of 0.21 ppm and 14.88 ppm and a quantitation limit of 0.24 ppm and 15.1 ppm were reported for thiram and dicyandiamide, respectively. The higher affinity of thiram towards BMNPs was probably due to the high affinity of the thiol group towards noble metals. The detection was achieved within 34 min, with negligible interferences of other contaminants. 

Core-shell Au@Ag BMNPs were also employed to reveal the presence of the herbicide diquat (as a cation) in apple juice [56]. BMNPs were prepared in different Au core size and Ag shell thickness, pointing out the influence of size on the performance as Raman active substrate. Only BMNPs with a diameter of 78 nm and 43 nm Au core showed an acceptable SERS enhancement effect, with a detection limit of 0.025 mg/L. Recently, Ngamaroonchote et al. prepared Au/Ag SERS substrate by using discarded blu-ray disc read only memory (BD-ROM) as starting material [57]. 

Adewoye et al. synthesized Au/Ag BMNPs from *Eichorniacrassipies*, an aquatic macrophyte, with a gold and silver weight composition of 8.18% and 19.55%, respectively [58]. The authors reported this eco-friendly approach provides BMNPs able to remove copper, zinc, lead and manganese from pharmaceutical effluent, with the higher affinity towards lead. 

## 5. Biological Activity Profile of Au/Ag BMNPs

The development of Au/Ag BMNPs has gained growing interest over the last few years (in view of their remarkable therapeutic potential) in particular as antimicrobial and anticancer agents. Likewise in Au/Ag BMNPs designed as biomedical materials or nanotechnological devices, size, shape, and surface chemical composition are key factors in determining the biological outcomes of the resulting BMNPs. Overall, small-sized and branched NPs are associated with superior biocidal and cytotoxic properties because of a larger surface area for the interaction with cellular, subcellular, and biomolecular targets [59]. The surface coat plays an essential role for the adhesion (primarily) to cell membranes and disruption of the latter and/or release of bioactive components into the cytoplasm. Then, as most bacteria and cancer cells possess an overall negative charge on their exterior surface (mainly due to the presence of peptidoglycan and sialic acid-rich glycoproteins, respectively) [60,61,62], BMNPs capped with positively charged moieties are more toxic than anionically-capped ones. Moreover, being that Ag is more toxic than Au as a cation, it is preferable to design Au/Ag nanostructures with the Au component as the inner part and the Ag component at the outer part as in the Au@Ag core-shell nanoarchitectures. The toxicity of the Ag component towards cells is most likely a cumulative effect of Ag^+^ ions and Ag NPs. Indeed, some authors showed that the toxicity is related to the release of Ag^+^ ions [63], while others have attributed the toxicity to the Ag NPs [64]. At a mechanistic level, Ag^+^ shows strong affinity towards the thiol (-SH) group, which is present is several biomolecules (such as enzymes and structural protein) that play a pivotal role in cellular homeostasis and replication. Interaction of the Ag^+^ ions with -SH groups inactivates such biomolecules, leading to a variety of effects including oxidative stress (as a consequence of a reduction of glutathione levels and increase of ROS levels), protein dysfunction, membrane damage, induction of apoptosis and autophagy, proteomic changes, and DNA inactivation [65,66]. Likewise Ag ions, Au ions (both in Au^+^ and Au^3+^ oxidation state) show affinity towards -SH group, and several Au ions included in organic complexes turned out to be effective as antimicrobial and/or anticancer agents [67,68]. However, in the Au NPs, the antibacterial and anticancer effects are most likely due to the compresence of other components within the nanosystems such as surface coating agents and bioactive molecules, such as those ones obtained through phytosynthesis [69]. Au NPs are mostly chosen for their extraordinary safety and biocompatibility profile, together with their unique optical (e.g., ability to be activated at relatively low radiation energy generating heat as in photothermal anticancer therapy) and surface properties (e.g., accessibility of use of surface modification agents for engineering strategies [70,71,72]. Hereafter, we report a few examples of the most recent research works dealing with Au/Ag BMNPs which possess noteworthy biological activity.

### 5.1. Antimicrobial Activity

In regard to the antimicrobial activity of Au/Ag BMNPs, the work of Fierascu et al. showed that Au/Ag BMNPs are more promising materials when compared to the related MNPs [33]. Although Ag NPs turned out be more effective against a wide panel of bacteria, fungi and yeasts (both in terms of MIC and MCBE—namely, minimal inhibitory concentration and minimal concentration of biofilm eradication, respectively), the bimetallic materials showed less mutagenic properties in the cytogenotoxicity test evaluated by means of *Allium cepa* L. root cells.

The fact that the Ag content (and eventually the Ag^+^ release) at the outermost layer of this type of bimetallic materials is directly related to the biocidal activity has been definitively confirmed by several recent works. For instance, Hu and co-workers proved this correlation by obtaining nanocomposites decorated with Au/Ag BMNPs using a cellulose dope as a generator for the one-pot synthesis of the NPs. Indeed, the nanocomposites with the best antimicrobial activity (evaluated against *E. coli* as a gram(-) and *S. aureus* as a gram(+)) and lower cytotoxicity (evaluated in COS-7 cells by MTT assay) turned out to be those ones containing an ultrathin Ag-rich outermost shell around an Au-rich core [73]. A similar outcome was obtained by Diem et al. who used dextran instead of cellulose as a support for the resulting monodispersed NPs. The antimicrobial activity in this case was evaluated against *Xanthomonas oryzae* pv. *oryzae* bacteria and *Magnaporthe grisea* fungi. Ag NPs and Au/Ag NPs with high content of Ag showed much higher activity than Au NPs. Besides, Au/Ag BMNPs showed a versatile tunability on the antimicrobial activity according to the Ag content at the surface of the NPs [74]. 

Bhanja et al. developed Au/Ag composite NPs endowed with moderate antibacterial activity (MIC = 85 μg/mL against *P. aeruginosa*) and huge antioxidant potential assessed in different free radical scavenging tests, including DPPH, NO· and H_2_O_2_ and the Fe^3+^ reducing assay [49]. This combined activity is another intriguing common feature of the Au/Ag BMNPs that can be further exploited for biomedical and industrial applications.

An example of Au@Ag core-shell NPs with antimicrobial potential has been reported by Ghosh and co-workers, in an eco-friendly synthesis which entails the use of *Dioscorea bulbifera* tuber extract as reducing and capping agent. Specifically, these NPs showed potent antibiofilm activity against *Acinetobacter baumannii* (~84%) and, to a lesser extent, against *P. aeruginosa* (~19%), *E. coli* (~22%) and *S. aureus* (~31%). Furthermore, these NPs showed remarkable antileishmanial activity (MIC = 32 μg/mL) assessed by the MTT method against the extracellular forms (promastigotes) of *Leishmania donovani*, the etiological agent of the visceral leishmaniasis in humans [75]. A more recent and exhaustive work dealing with this tropical neglected infectious disease has been reported by Alti et al. In this case the authors used fenugreek, coriander, and soybean leaf extracts for the green synthesis of three different types of Au/Ag BMNPs. All of them exhibited high antileishmanial activity against both promastigote and amastigoste stages (intracellular form) of *L. donovani*. In particular, the IC_50_ values against promastigotes were found in the range 0.03–0.035 μg/mL, which is much lower than the reference drug miltefosine (IC_50_ = 10 μg/mL). The amastigotes instead were reduced by 31–46% in macrophages. Moreover, these Au/Ag BMNPs were able to promote ROS formation and induce apoptosis in promastigotes, a factor that could further potentiate the antileishmanial activity against the intracellular forms of the parasite [24].

### 5.2. Anticancer Activity 

In the potential use of Au/Ag BMNPs for anticancer therapy, the Au component of the nanosystems usually plays a dominant role in view of the unique properties of the Au NPs ranging from safety and biocompatibility to responsivity to physicochemical stimuli that make them suitable for both diagnosis and treatment of different types of tumors [70,71,76,77,78]. An outstanding work in this regard was carried out by Chen et. al. which prepared thorny star-shaped Au/Ag BMNPs capped with modified chitosan (*O*-carboxymethylchitosan, labelled with fluorescein isothiocyanate in order to investigate the cancer cells internalization by confocal microscopy) for photothermal therapy [79]. The peculiarity of these hybrid nanosystems is that the wavelengths absorbed can be tuned from visible light to near infrared (NIR) by controlling their shape, which in turn can be tuned by changing the ratio of the metal precursors in the synthetic process. The cell viability of the Au/Ag BMNPs was assessed in three cell lines—namely, oral mucosa fibroblasts (OMF), oral epithelia cells (S-G), and oral cancer cells (SAS), wherein the NPs turned out to be non-toxic towards all three cell lines and able to promote an effective photothermal ablation of the SAS cancerous cells under NIR irradiation [79]. Another perspective work for anticancer radiotherapy which entails the employment of Au/Ag BMNPs was carried out by Ahmed et al. They obtained Au@Ag core-shell BMNPs with optimal radio-sensitizing activity in the colloid form under cobalt-60 radiation that could be exploited in the modern cancer radiotherapy upon direct injection into the tumor tissue [41]. An Au@Ag core-shell architecture for the development of BMNPs was proposed also by Hada et al. (see hereinabove). In this case the huge potential for cell imaging and cancer diagnosis of these materials relies on their use as SERS-nanotags with ultra-bright traceability and pH sensitivity inside cells, specifically assessed inside human ovarian adenocarcinoma cells NIH:OVCAR-3. The double chitosan coating (for the Au core and for the Ag shell) plays a fundamental role in empowering the BMNPs with high stability and biocompatibility, and, at the same time, in forestalling antiproliferative effects towards cells [31]. Au-Ag alloy BMNPs, endowed with cytotoxic activity instead, were obtained by Botha et al. through a standard green synthetic method which entails the use of an extract of golden rod (*Solidago canadensis*) as a reducing and stabilizing agent. The antiproliferative effects of these BMNPs were evaluated against two cancerous cell lines, i.e., HuTu-80 (human intestinal) cells and H4IIE-*luc* (rat hepatoma) cells. BMNPs were more cytotoxic than the two monometallic counterparts under the same experimental conditions, and, since the Au NPs did not exhibit any toxic effect, the functional role in the uptake of NPs by cells of the Au component of the material has been reasonably assumed [80]. BMNPs with alloyed structure and considerable anticancer properties were also obtained by Cholula-Díaz’s research group (see hereinabove within the green synthesis section) [26]. In this case, the cytotoxic activity range was 10–20 μg/mL against human melanoma cells (ATCC) with no effects towards healthy cells (human dermal fibroblasts; HDF). Besides, these Au-Ag BMNPs showed promising antibacterial activity against multidrug-resistant (MDR) *E. coli* (IC_50_ = 4.92 μg/mL) and methicillin-resistant *S. aureus* (MRSA; IC_50_ = 6.95 μg/mL).

## 6. Conclusions

In this review, the more representative literature examples of green synthesis for the preparation of Au/Ag BMPNs with different chemical arrangements (i.e., alloyed structure, core-shell, hetero-structured, etc.) together with their recent research progress for applications in catalytic, electrochemical, sensing, and environmental remediation fields, have been surveyed. Particular attention was paid to the analyses of biological profiles of BMNPs and their potential applications as antimicrobial and antitumor agents. 

The complete characterization of BMNPs, should be considered an essential prerequisite for any applications, and it should include not only the determination of the classical parameters that are required to characterize MNPs (i.e., size, size distribution, shape, crystallographic nature, surface functionalization, and charge), but also further information regarding the overall elemental composition, the ratio of the two metals, and the internal distribution and homogeneity of the elements within individual nanoparticles. However, despite promising developments and a considerable amount of work in this field, often only fragmentary data have been reported for the characterization of BMNPs produced by phytosynthesis. Looking at the unique optical, thermal, electrical and biological properties of BMNPs and at the opportunity of their technology, we believe that future efforts will be devoted to overcoming the current limitations of green approaches for BMNP synthesis. 

## Figures and Tables

**Table 1:** The main representative examples of green procedures reported for the preparation of BMNPs as alloy, nanocomposite, and core-shell.

**Reference**	**BMNPs**	**Matrix**	**BMNP Features**	**BMNP Application**
[24]	Au-Ag alloy	fenugreek, soybean, and coriander leaf extracts	monodispersed, spherical, sizes of 10−12 nm, LSPR at 522–541 nm.	antileishmanial activity
[25]	Au-Ag alloy	*Lawsonia inermis* seed aqueous extracts	polygonal, spherical and irregular shaped, sizes 15–35 nm, LSPR at 537 nm.	photocatalytic reduction/degradation against 4-nitrophenol and methyl orange dye
[26]	Ag-Au alloy	potato starch	icosahedral (quasi-spherical), size of 9.7 nm, LSPR at λm = 463 nm	antimicrobial activity, anticancer effect on human melanoma cells
[27]	gold-based anisotropic nanostructures	starch-capped silver/gold alloy as seeds and H_2_O_2_	dimers, trimers, tetramers, quasi-spherical NPs (size of 22.5 nm), LSPR at λm = 522 nm; λm = 7 02 nm	SERS substrates
[28]	Au/Ag BMNPs nanocomposites	kondagogu rubber (GK)	spherical, size of 23 nm, λ_m_ = 711 nm	catalytic reduction of 4-nitrophenol
[29]	Ag@Au core shell	callus extract of *Lithospermum erythrorhizon*	spherical and elliptical shaped, 3–451 nm. LSPR at λm = 545 nm	cytotoxic properties
[30]	Au@Ag@AgCl core double shells	leaves extract of *Momordica charantia*	average size of 30–35 nm or 20–25 nm. LSPR at λm = 400 nm; λm = 530 nm	wastewater treatment
[31]	Au@Ag core-shell	chitosan	spherical average size of 29 nm LSPR at λm = 409 nm	SERS nanotag (cell imaging)
[32]	Au@Ag and Ag@Au core-shell	cyclodextrin	spherical, size of 15 nm. LSPR at λm = 413 nm (Au@Ag); λm = 495 nm (Ag@Au)	radical scavenging property
[33]	Au/Ag nanoarchitectures	ethanolic extract of *M. officinalis*	flower-like structure, size 8 nm, LSPR at λm = 541 nm; λm = 413 nm	antimicrobial properties.
[34]	Au-Ag alloy nanoclusters (AuAgNCs)	bovine serum albumin (BSA)	core size < 2 nm	photodynamic therapy and bioimaging

## Data Availability

Data sharing is not applicable to this article.

## Abbreviations

NPs	nanoparticles
BMNPs	bimetallic nanoparticles
MNPs	monometallic nanoparticles
GRR	galvanic replacement reaction
LSPR	Localized Surface Plasmon Resonance
Me	metal
GK	kondagogu rubber
BK-39	Lithospermum erythrorhizon
CDs	cyclodextrin
NCs	nanoclusters
BSA	bovine serum albumin
SAXS	small-angle X-ray scattering
TEM	transmission electron microscopy
ICP-MS	inductively coupled plasma mass spectrometry
MEF	metal-enhanced fluorescence
LA	lactose
LB	lactobionic acid
CIP	Ciprofloxacin
MIC	minimal inhibitory concentration
MCBE	minimal concentration of biofilm eradication
SERS	Surface Enhanced Raman Scattering
